# Bioluminescence Imaging of *Chlamydia muridarum* Ascending Infection in Mice

**DOI:** 10.1371/journal.pone.0101634

**Published:** 2014-07-01

**Authors:** Jessica Campbell, Yumeng Huang, Yuanjun Liu, Robert Schenken, Bernard Arulanandam, Guangming Zhong

**Affiliations:** 1 Department of Microbiology and Immunology, The University of Texas Health Science Center at San Antonio, San Antonio, Texas, United States of America; 2 Department of Obstetrics and Gynecology, The University of Texas Health Science Center at San Antonio, San Antonio, Texas, United States of America; 3 Department of Biology, University of Texas at San Antonio, San Antonio, Texas, United States of America; Midwestern University, United States of America

## Abstract

Chlamydial pathogenicity in the upper genital tract relies on chlamydial ascending from the lower genital tract. To monitor chlamydial ascension, we engineered a luciferase-expressing *C. muridarum*. In cells infected with the luciferase-expressing *C. muridarum*, luciferase gene expression and enzymatic activity (measured as bioluminescence intensity) correlated well along the infection course, suggesting that bioluminescence can be used for monitoring chlamydial replication. Following an intravaginal inoculation with the luciferase-expressing *C. muridarum*, 8 of 10 mice displayed bioluminescence signal in the lower with 4 also in the upper genital tracts on day 3 after infection. By day 7, all 10 mice developed bioluminescence signal in the upper genital tracts. The bioluminescence signal was maintained in the upper genital tract in 6 and 2 mice by days 14 and 21, respectively. The bioluminescence signal was no longer detectable in any of the mice by day 28. The whole body imaging approach also revealed an unexpected airway infection following the intravaginal inoculation. Although the concomitant airway infection was transient and did not significantly alter the genital tract infection time courses, caution should be taken during data interpretation. The above observations have demonstrated that *C. muridarum* can not only achieve rapid ascending infection in the genital tract but also cause airway infection following a genital tract inoculation. These findings have laid a foundation for further optimizing the *C. muridarum* intravaginal infection murine model for understanding chlamydial pathogenic mechanisms.

## Introduction

Pelvic Inflammatory Diseases can be caused by ascending infection from sexually transmitted microorganisms, including *Chlamydia trachomatis* [1], [2]. However, it remains unclear how *C. trachomatis* organisms ascend to the upper genital tract. Although live chlamydial organisms or chlamydial nucleic acids can be monitored from women's vaginal/cervical swab samples [3], these approaches only measure the lower genital tract infection. Due to the difficulty in routinely detecting upper genital tract infection in women, it is not possible to study *C. trachomatis* ascension mechanisms in humans. *Chlamydia muridarum* organisms have been extensively used to model the mechanisms of *C. trachomatis* pathogenesis [4]. This is because intravaginal inoculation with *C. muridarum* in mice can lead to ascending infection, resulting in upper genital tract pathology that is similar to what is induced by *C. trachomatis* in humans [5]. During mouse model studies, live organism recovery from vaginal swabs is often used for monitoring chlamydial infection in the lower genital tract [6], [7]. Some have monitored ascending infection in mice by euthanizing mice for examining infection status in the upper genital tract tissues at multiple time points after infection [6], [8], [9]. This approach not only requires euthanizing many mice but also fails to follow the same animals for progression of ascending infection and eventual development of upper genital tract pathology.

Bioluminescence imaging with firefly luciferase has been used to monitor viral and bacterial infections in live animals [10], [11], [12]. This technology allows one to continuously follow live microbial infection in the same animals over time since luciferase is expressed by the organisms that are being followed. Transformation of chlamydial organisms has become possible [13], [14], [15]. We recently developed a protocol for transforming *C. muridarum* [16], which has made it possible for utilizing the luciferase-catalyzed bioluminescence imaging technology for monitoring *C. muridarum* infection in mice.

In the current study, we engineered a luciferase-expressing *C. muridarum* strain and found that the luciferase enzymatic activity measured as bioluminescence intensity correlated well with *C. muridarum* replication in cell culture system. When the luciferase-expressing *C. muridarum* were inoculated intravaginally into mice, bioluminescence signal was detected from the same mice along the entire course of infection. This technology has enabled us to monitor *C. muridarum* spreading from the lower to upper genital tracts in real time, which has laid a foundation for further understanding chlamydial pathogenic mechanisms.

## Materials and Methods

### 1. Construction of recombinant plasmid for transformation

A DNA sequence from *C. muridarum* plasmid pgp4 promoter region was linked to the firefly luciferase gene *luc* by PCR. The firefly luciferase gene was amplified from pGME-luc vector purchased from Promega (Madison, WI). The transcription termination signal sequence stemming from ORF CT579 of *Chlamydia trachomatis* serovar D was conjoined to the 3 prime of the luciferase gene. In this way, an independent operon consisting of pgp4 promoter, *luc* gene and termination signal was established and inserted in front of the GFP-CAT gene of the pGFP::CM plasmid shuttle vector using an infusion cloning technique as described previously [16]. The newly created recombinant plasmid pGFP-luc-CM was used for transforming *C. muridarum* as described below.

### 2. Transforming plasmid-free C. muridarum organisms

The pGFP-luc-CM plasmid was introduced into a plasmid-free *C. muridarum* strain designated as CMUT3 [9] in the form of a purified elementary body (EB) by following the protocol published previously [15], [16]. The luciferase-expressing *C. muridarum* organisms were plaque-purified as described previously [17], [18] for both *in vitro* characterization and *in vivo* imaging as described below.

### 3. Detecting luciferase activity by measuring bioluminescence generated from luciferase-catalyzed luciferin

HeLa cells grown in 24-well plate were infected with luciferase-expressing *C. muridarum* organisms at an MOI of 2.5. At 6 h, 9 h, 12 h, 15 h, 18 h, 21 h, 24 h, 27 h & 30 h after infection, D-luciferin (cat#P1043, Promega) was added to each well at the final concentration of 1 mg/ml/well. The bioluminescence intensity from each well was detected and analyzed using Spectra MAX GEMINXS and SOFTmax Pro software (Molecular Devices, Sunnyvale, CA). After bioluminescence measurement, the infected cells were harvested using TRIzol reagent (Life Technologies, Grand Island, NY) for quantitating luciferase mRNA using quantitative RT-PCR as described below.

### 4. Quantitative real-time RT-PCR

The culture samples harvested in TRIzol reagent as described above were extracted for total RNA using a Direct-zol RNA Miniprep kit (Zymo Research, Irvine, CA). The total RNA was used as template for reverse transcription to produce cDNAs with random hexamer primers with a ThermoScript reverse transcription-PCR (RT-PCR) system (Life Technologies, Grand Island, NY). The luciferase cDNA copy numbers were quantitated using a TaqMan RT-PCR assay carried out on a CFX96 Touch Deep Well real-time PCR detection system (Bio-Rad, Hercules, CA) with iQ Supermix (Bio-Rad). The following primers were used: *luc* forward 5′-GAACATCACGTACGCGGAATA-3′ and reverse 5′-CCAACACCGGCATAAAGAATT-3. The probe sequence used was 5'-/56-FAM/TCG GTT GGC/ZEN/AGA AGC TAT GAA ACGA/3IABkFQ/-3'. The qPCR conditions included an initial denaturation step at 95°C for 3 minutes, followed by 40 cycles of amplification at 95°C for 15 seconds and 60°C for 1 minute.

### 5. Animal infection

The luciferase-expressing *C. muridarum* used in the current study were propagated in HeLa cells (human cervical carcinoma epithelial cells, ATCC cat# CCL2.1), purified, aliquoted and stored as described previously [19]. Female Balb/cJ (000651) were purchased at the age of 5 to 6 weeks old from Jackson Laboratories (Bar Harbor, Maine). Each mouse was inoculated intravaginally with 2×10^5^ IFUs of live luciferase-expressing *C. muridarum* in 20 µl of SPG (sucrose-phosphate-glutamate buffer). The animal experiments were carried out in accordance with the recommendations in the Guide for the Care and Use of Laboratory Animals of the National Institutes of Health. The protocol was approved by the Committee on the Ethics of Laboratory Animal Experiments of the University of Texas Health Science Center at San Antonio.

### 6. Monitoring live C. muridarum organism recovery from vaginal swabs

To monitor live organism shedding, vaginal swabs were taken on different days after infection. Each swab was suspended in 500 µl of ice-cold SPG followed by vortexing with glass beads, and the released organisms were titrated on HeLa cell monolayers in duplicates as described previously [20]. The total number of IFUs per swab was calculated based on the number of IFUs per field, number of fields per coverslip, dilution factors and inoculation and total sample volumes. An average was taken from the serially diluted and duplicate samples for any given swab. The calculated total number of IFUs/swab was converted into log_10_ and the log_10_ IFUs were used to calculate means and standard deviations for each group at each time point.

### 7. In vivo imaging

Mice infected intravaginally infected with luciferase-expressing *C. muridarum* organisms were imaged using the Xenogen IVIS imaging system (PerkinElmer, Hopkinton, MA) on days 3, 7, 10, 14, 21 and 28 after infection when vaginal swabs were taken. Prior to imaging, 500 µl of D-luciferin (40 mg/ml in sterile PBS) were intraperitoneally injected into each mouse. Twenty-five minutes after injection, mice were anesthetized in an induction chamber with 2% isofluorane. Bioluminescence images of the whole mouse bodies were captured. To acquire high quality images, the exposure time was set as 1 minute for each image. To measure photon radiance quantitatively, regions of interest (ROI) were pre-selected on the surface of the mouse bodies using the automatic ROI. Intensity of bioluminescence was analyzed by using a Living Image software from PerkinElmer.

### 8. Statistical analyses

The Spearman Correlation was used to analyze relationships between the different parameters, including luciferase gene expression versus luciferase activity (bioluminescence intensity) along the infection course in cell culture and live organisms recovered from the lower genital tract versus bioluminescence intensity taken from either the lower or upper genital tract areas. The statistical significance of these correlations was analyzed using Student *t*-Test.

## Results

### 1. Characterization of luciferase-expressing C. muridarum

**Figure 1:**
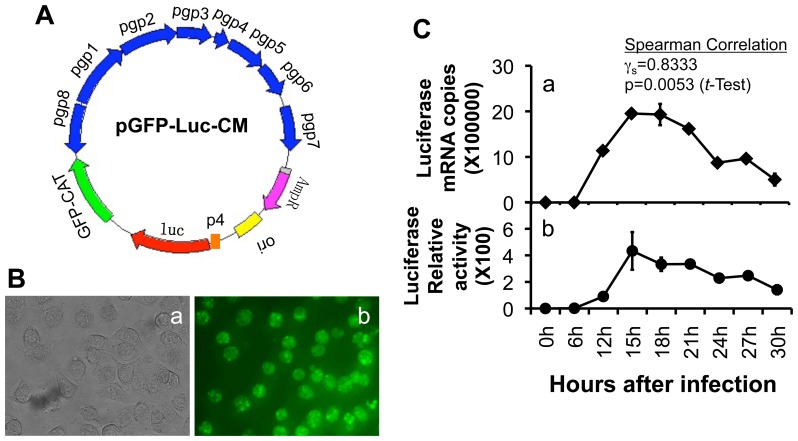
Construction and characterization of luciferase-expressing C. muridarum. (A) The DNA fragment consisting of pgp4 promoter (p4, orange), luciferase gene (luc, red) and transcriptional termination signal (not shown) was inserted in front of the GFP-CAT gene (green) of the pGFP::CM shuttle vector to create a new recombinant plasmid pGFP-luc-CM. (B) The newly created recombinant plasmid pGFP-luc-CM was used for transforming a plasmid-free *C. muridarum* organism to produce a plasmid-competent and luciferase-expressing *C. muridarum* (grey inclusions in panel a and green in b). (C) HeLa cells infected with the luciferase-expressing *C. muridarum* organisms were used for both measuring luciferase transcripts using qRT-PCR (panel a) and detecting luciferase activity by quantitating intensity of bioluminescence generated from luciferase-catalyzed luciferin (panel b). Note that both luciferase gene expression and activity became detectable at 12, peaked at 15 and significantly decreased by 24 hours after infection, with a coefficient (r_s_) of 0.8333 and p value of 0.0053 (student *t*-Test).

The luciferase-expressing *C. muridarum* were used to infect HeLa cells for monitoring both the luciferase gene expression and luciferase activity measured as bioluminescence intensity (Fig. 1). The luciferase expression level correlated well with the luciferase activity. Both became detectable at 12 hours (h) after infection. By 15 h after infection, both reached peak levels. Significant decrease was noted for both by 24 hours after infection. This time course is consistent with the rapid *C. muridarum* replication cycle in which the reticulate body (RB) replication peaks between 15 and 18 h with most RB differentiating into elementary body (EB) by 24 h after infection. These observations suggest that luciferase activity can be used for monitoring *C. muridarum* replication.

### 2. The in vivo imaging reveals rapid C. muridarum ascending infection with peak levels of ascension within the first 10 days after intravaginal inoculation

**Figure 2:**
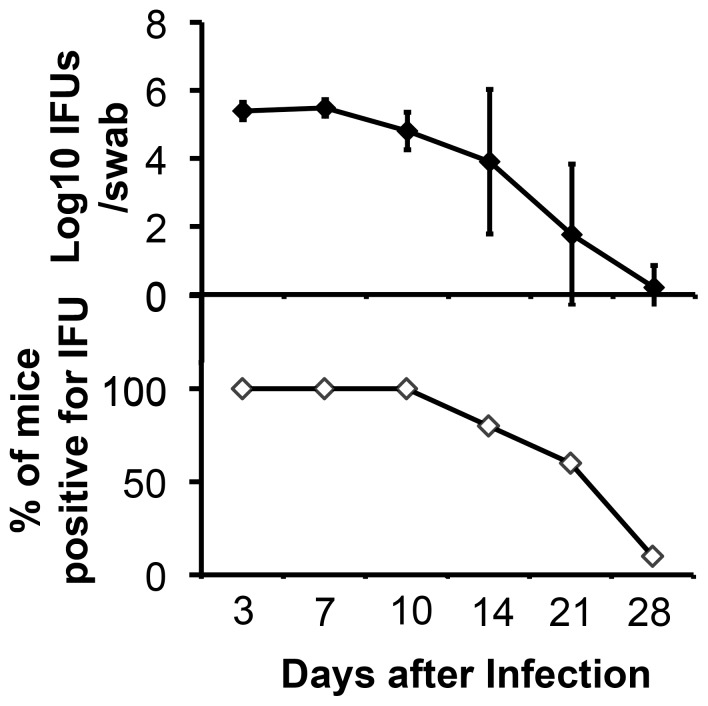
Live C. muridarum organism recovery from vaginal swabs along the infection time course. After the intravaginal infection with the luciferase-expressing *C. muridarum* organisms, vaginal swabs were taken at different time points as indicated along the X-axis for monitoring live chlamydial organism shedding from the lower genital tract. The number of live organisms recovered from the vagina/cervix swabs expressed as log_10_ IFUs per swab (top panel) and percent of mice that remained positive for shedding live organisms (bottom panel) were displayed along the Y-axis. Note that high levels of live organisms were recovered from the lower genital tract during the first 10 days after infection and the shedding dropped significantly by day 14 after infection, which is consistent with the time courses of genital tract infection with wild type *C. muridarum*.

**Figure 3:**
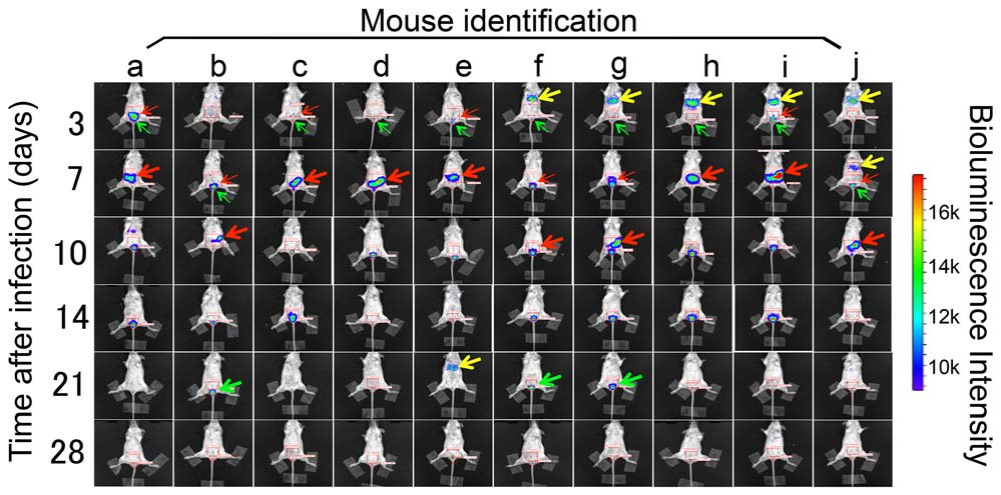
In vivo imaging of intravaginal infection with luciferase-expressing C. muridarum in mice. The 10 female Balb/cJ mice labeled “a” to “j” as indicated on top of the figure were intravaginally infected with luciferase-expressing *C. muridarum* as described in Fig.2 legend. At each time point after infection (when vaginal swabs were taken, see Fig. 2 legend) as indicated along left side of the figure, all mice were subjected to whole body in vivo imaging. The relative bioluminescence intensity was shown in the form of color heat map as indicated along the right side of the figure. The intensity of bioluminescence was also quantitatively measured from the pre-defined lower and upper genital tract areas. Significant bioluminescence signals first becoming detectable in the lower (green) or upper (red) genital tract areas were marked with thin arrows. Peak bioluminescence signals in the upper genital tract of each mouse were marked with thick red arrows. Bioluminescence signals remained detectable in the genital tract areas on day 21 after infection were indicated with think green arrows while those in the chest areas with thick yellow arrows. Note that most mice achieved a maximal ascending infection by days 7 or 10 after intravaginal infection while some mice have also acquired airway infection.

**Figure 4:**
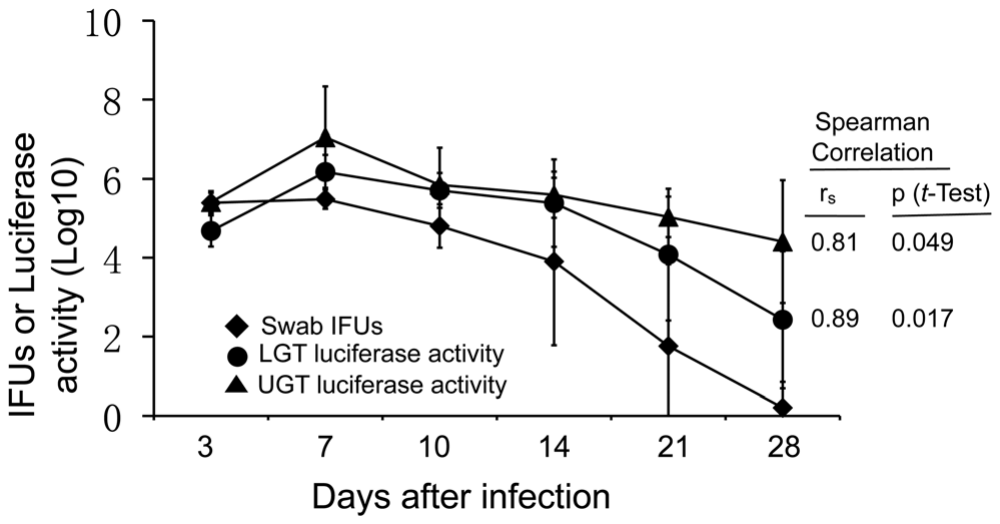
Correlation of live organism recovery with bioluminescence intensity. The live organisms recovered from the lower genital tract (swab IFUs, diamond) was analyzed against bioluminescence intensity or luciferase activity measured in the lower (circle) or upper (triangle) genital tracts (both in log_10_ shown along Y-axis) along the entire infection course after intravaginal inoculation (X-axis) using Spearman Correlation followed by student *t*-Test. Note that a correlation coefficient (r_s_) of 0.81 with a p value of 0.049 (*t*-Test) was found between the IFUs and the upper genital tract bioluminescence while 0.89 with p = 0.017 found between the IFUs and the lower genital tract bioluminescence.

To monitor ascending infection, 10 female Balb/cJ mice were intravaginally infected with luciferase-expressing *C. muridarum* and monitored by both titrating live organism recovery from the lower genital tract and imaging whole mice for bioluminescence signals. Significant amounts of live organisms were recovered from the lower genital tracts of all 10 mice during the first 10 days after infection (Fig. 2). Starting day 14, both the level of live organisms and number of mice with positive shedding significantly decreased. By day 28, all but one mouse cleared infection. This time course is consistent with those of genital tract infection with wild type *C. muridarum* [9], [21], suggesting that luciferase expression did not significantly alter *C. muridarum* infection in the mouse genital tract. As shown in Fig. 3, the simultaneously monitoring for luciferase-generated bioluminescence using the Xenogen IVIS imaging system revealed that as early as 3 days after infection, bioluminescence signal became detectable in the upper genital tract area of 4 mice, suggesting rapid ascension by *C. muridarum* organisms upon intravaginal inoculation. By day 7, all 10 mice displayed bioluminescence signal in the upper genital tract area with peak levels of the signal in 6 mice. The remaining 4 mice developed peak levels of bioluminescence signal in their upper genital tract by day 10. These observations indicated that all mice developed peak ascending infection within the first 10 days after intravaginal inoculation. The ascending infection was maintained in the upper genital tract in 6 and 2 mice by days 14 and 21, respectively. No more bioluminescence signal was detected in any mice by day 28. When the relationships between live organisms recovered from the lower genital tract and bioluminescence intensity measured from either the lower or upper genital tract areas were analyzed (Fig. 4), significant correlations were found. Interestingly, the live organism recovery from the lower genital tract displayed a stronger correlation with the lower than upper genital bioluminescence signals.

### 3. The whole body in vivo imaging also reveals an unexpected airway co-infection following an intravaginal inoculation with C. muridarum

**Figure 5:**
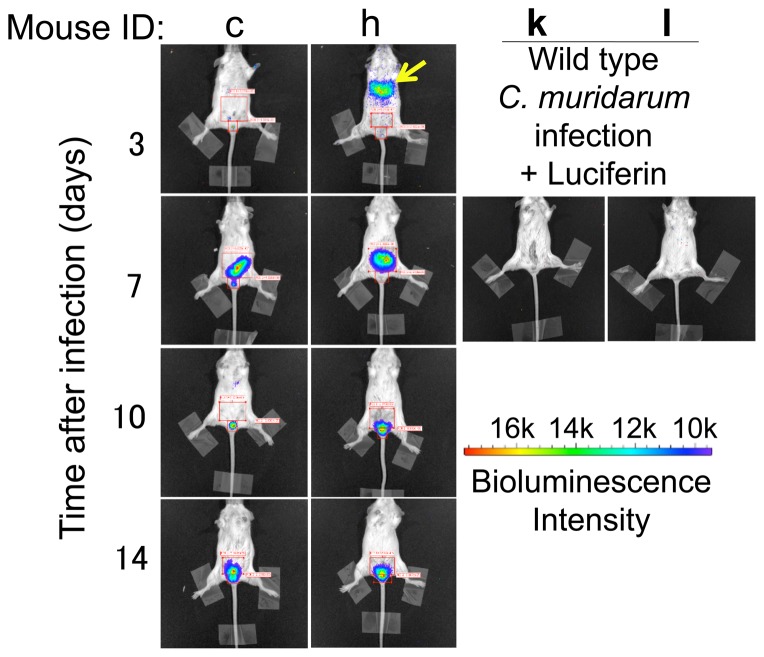
Enlarged bioluminescence images from mice with or without airway signals and mice infected with wild type C. muridarum. The bioluminescence images from mice c and h shown in Fig. 3 were enlarged and shown in the same order of infection time as in Fig. 3. Airway bioluminescence signal in mouse h was marked with a yellow arrow. Mice k and l were infected with wild type *C. muridarum* and 7 days after infection, bioluminescence imaging was taken after injection with luciferin. Note a similar genital tract infection time course between mice c and h and lack of any significant bioluminescence signals in mice k and l.

To our surprise, all 5 mice housed in the same cage displayed very strong bioluminescence signals in the chest area on day 3 after intravaginal infection (Fig. 3, yellow arrows). However, the signals were transient and completely disappeared in 4 mice by day 7. The remaining mouse cleared the signal in the chest area by day 10. These observations suggest that mice can acquire respiratory infection from intravaginally inoculated organisms. One mouse (labeled as mouse “e”) in the other cage also acquired a transient airway infection on day 21 after intravaginal inoculation while 4 of the 5 mice, including mouse “e”, cleared genital tract infection on the same day. The remaining mouse with positive genital tract bioluminescence signal in the same cage was labeled as mouse “b”. The bioluminescence signal from mouse “b” was mainly localized in the lower genital tract area, suggesting that mouse e inhaled chlamydia shed from mouse b. This interpretation seemed to be more likely than the alternate explanation of hematogenous dissemination from the genital tract to the lung because the lung infection occurred only in co-caged mice. It is worth noting that the transient airway infection did not seem to significantly alter the genital tract infection time courses. For example, mice “g”, “h” & “I”, which acquired airway infection on day 3, displayed similar genital tract infection time courses with mice “b”, “c” & “a”, which never acquired detectable airway infection. To further clarify the above point, the images from mice c and h were enlarged (Fig. 5), which clearly showed that mice c and h developed a similar genital tract infection course regardless of their airway infection status. As negative controls, mice k and l that were infected with wild type *C. muridarum* developed no detectable bioluminescence signals even after injection with luciferin. It is thus clear that any significant bioluminescence signal detected in the mice infected with luciferase-expressing *C. muridarum* must come from the catalysis of luciferin by luciferase.

## Discussion

By imaging bioluminescence signals catalyzed by *Chlamydia muridarum*-expressed luciferase, we have successfully visualized *Chlamydia muridarum* organisms in mouse genital tract without euthanizing any mice. This non-invasive approach has allowed us to conclude that *C. muridarum* organisms can start ascension as early as day 3 and maximize ascension on days 7 or 10 after intravaginal inoculation in female Balb/cJ mice. Please also note a considerable variation in both chlamydial ascending time courses and ascending patterns among the mice of the same inbred strain. For example, mouse “a” developed a very significant ascending infection on day 3 and the ascending peaked in the left uterine horn and oviduct on day 7 while mouse “b” in the same cage did not start ascension until day 7 and the chlamydial organisms reached a peak level of ascending in the right side of the genital tract by day 10 after infection. The delayed ascending infection in mouse “b” seemed to enable mouse “b” to develop longer time course of bioluminescence signal in the genital tract. Mice “f” & “g” with delayed ascending peaks on day 10 also developed prolonged genital tract infection. The question is whether these variations can lead to differences in development of upper genital pathology.

Unexpectedly, the whole body imaging also revealed significant bioluminescence signals in the chest area, indicating airway infection. Although all mice were only intravaginally inoculated with *C. muridarum* organisms, significant lung infection developed. All mice with lung infection on day 3 after intravaginal inoculation were housed in the same cage. We speculated that the airway infection was caused by inhaling luciferase-expressing *C. muridarum* leaked from mouse vagina. We did not independently detect live organisms from the lungs that displayed bioluminescence signal. This is because we wanted to keep the mice alive and monitor the same mice over time. Nevertheless, we expect to detect live organisms from these lungs. This speculation is consistent with previous findings that live organisms were recovered from lung tissues of mice intravaginally infected with *C. muridarum* [22], [23]. It is also possible that the lung bioluminescence signal might come from nonviable materials containing luciferase activity. This hypothesis is consistent with the observation that the bioluminescence signal from the chest area was often transient. The airway signal detected on day 3 resolved by day 7 in most mice and residual airway signal was visible only in one mouse by day 7. More importantly, mice with lung bioluminescence signal did not show any significant alteration in the genital tract infection course. Mice with or without airway signal all developed peak ascending infection in the genital tract on either day 7 or 10 after infection. Nevertheless, a prolonged airway infection may significantly alter infection courses in the genital tract. For example, mouse “j” displayed airway infection on both days 3 & 7, which correlated with the shortened time course of genital tract infection. Mouse “j” cleared genital tract infection by day 14 when bioluminescence signals were still detectable in the genital tract of most other mice. This observation is consistent with the hypothesis that active lung infection can induce robust adaptive immunity that may shorten the infection course in the genital tract. This hypothesis is supported by a recent report that an intranasal inoculation with 50 IFUs of live *C. muridarum* induced sterile immunity in the genital tract against a challenge infection with 2×10^5^ IFUs [24]. Thus, the airway bioluminescence signals should be carefully examined and properly interpreted.

Interestingly, we did not detect any significant bioluminescence signal from the mouse gastrointestinal tract despite a previous report that live organisms were recovered from the gastrointestinal tract following an intravaginal inoculation with *C. muridarum* [23]. In any case, we will be looking out the possibility using the whole body imaging technology since it was recently reported that *C. muridarum* could survive in the gastrointestinal tract for long periods of time, which could serve as a reservoir for persistent infection [25].

We are aware that the bioluminescence signals detected may not always correlate with chlamydial biosynthesis and replication in genital tract tissues. This is because bioluminescence is generated by luciferase-mediated catalysis of D-luciferin and luciferase expression controlled by pgp4 promoter may not always be proportional to chlamydial replication. Many factors independent of chlamydial replication may affect the luciferase-catalyzed bioluminescence intensity, including the expression level of luciferase and plasmid copy number. Although we have demonstrated a strong association of bioluminescence intensity with luciferase expression and chlamydial replication in cell culture system, it is not known whether such an association can be maintained during *C. muridarum* infection *in vivo*. We have previously shown that the plasmid copy number of the *C. muridarum* transformants is 2 to 3 fold higher than that of wild type *C. muridarum* [16]. The luciferase-expressing C. muridarum is no exception and its plasmid copy number is also 2.5 fold higher than that of wild type *C. muridarum* (data not shown). However, it is not known whether the plasmid copy number can be maintained at a constant level during *C. muridarum* infection in mice. Nevertheless, the plasmid copy number in *C. trachomatis* infecting human ocular tissues seemed to be relatively stable [26]. More importantly, although it is certain that lack of plasmid can significantly attenuate the pathogenicity of both *C. muridarum* and *C trachomatis* [9], [27], the effect of plasmid copy number on chlamydial pathogenicity remains unclear [26]. In any case, direct detection of chlamydial organisms in the upper genital tract may still be necessary for validating the infection status. The fact that a stronger correlation of the lower genital tract live organism recovery was established with the lower than the upper genital tract bioluminescence signals suggests that bioluminescence intensity can be used to both track live chlamydial organisms and more accurately detect upper genital tract infection. Thus, the luciferase imaging technology may have provided a convenient means for both investigating chlamydial pathogenic mechanisms and evaluating efficacies of intervention and prevention strategies.
